# Validity of a two-component imaging-derived disease activity score for improved assessment of synovitis in early rheumatoid arthritis

**DOI:** 10.1093/rheumatology/kez049

**Published:** 2019-03-01

**Authors:** Elizabeth M A Hensor, Paul McKeigue, Stephanie F Ling, Marco Colombo, Jennifer H Barrett, Jackie L Nam, Jane Freeston, Maya H Buch, Athina Spiliopoulou, Felix Agakov, Stephen Kelly, Myles J Lewis, Suzanne M M Verstappen, Alexander J MacGregor, Sebastien Viatte, Anne Barton, Costantino Pitzalis, Paul Emery, Philip G Conaghan, Ann W Morgan

**Affiliations:** 1 Leeds Institute of Rheumatic and Musculoskeletal Medicine, School of Medicine, University of Leeds; 2 NIHR Leeds Biomedical Research Centre, Leeds Teaching Hospitals NHS Trust, Leeds; 3 Usher Institute for Population Health Sciences and Informatics, University of Edinburgh, Edinburgh; 4 Arthritis Research UK Centre for Genetics and Genomics, Division of Musculoskeletal and Dermatological Sciences, The University of Manchester, Manchester; 5 Leeds Institute of Cancer and Pathology, School of Medicine, University of Leeds, Leeds; 6 Pharmatics Limited, Edinburgh; 7 William Harvey Research Institute, Barts and the London School of Medicine and Dentistry, Queen Mary University of London, London; 8 Arthritis Research UK Centre for Epidemiology, Division of Musculoskeletal and Dermatological Sciences, The University of Manchester, Manchester; 9 NIHR Manchester Biomedical Research Centre, Manchester University NHS Foundation Trust, Manchester; 10 Norwich Medical School, University of East Anglia, Norwich, UK

**Keywords:** rheumatoid arthritis, DAS28, ultrasonography, synovitis

## Abstract

**Objectives:**

Imaging of joint inflammation provides a standard against which to derive an updated DAS for RA. Our objectives were to develop and validate a DAS based on reweighting the DAS28 components to maximize association with US-assessed synovitis.

**Methods:**

Early RA patients from two observational cohorts (*n* = 434 and *n* = 117) and a clinical trial (*n* = 59) were assessed at intervals up to 104 weeks from baseline; all US scans were within 1 week of clinical exam. There were 899, 163 and 183 visits in each cohort. Associations of combined US grey scale and power Doppler scores (GSPD) with 28 tender joint count and 28 swollen joint count (SJC28), CRP, ESR and general health visual analogue scale were examined in linear mixed model regressions. Cross-validation evaluated model predictive ability. Coefficients learned from training data defined a re-weighted DAS28 that was validated against radiographic progression in independent data (3037 observations; 717 patients).

**Results:**

Of the conventional DAS28 components only SJC28 and CRP were associated with GSPD in all three development cohorts. A two-component model including SJC28 and CRP outperformed a four-component model (*R*^2^ = 0.235, 0.392, 0.380 *vs* 0.232, 0.380, 0.375, respectively). The re-weighted two-component DAS28CRP outperformed conventional DAS28 definitions in predicting GSPD (Δtest log-likelihood <−2.6, *P* < 0.01), Larsen score and presence of erosions.

**Conclusion:**

A score based on SJC28 and CRP alone demonstrated stronger associations with synovitis and radiographic progression than the original DAS28 and should be considered in research on pathophysiological manifestations of early RA. Implications for clinical management of RA remain to be established.

**Rheumatology key messages**

- Tender joint count and general health visual analogue scale are not associated with imaging-detected inflammation.
- A two-component DAS28 shows closer association with inflammation and structural damage than the original score.
- This new score should be considered in research on pathophysiological manifestations of early RA.

## Introduction

The DAS, introduced in 1990 [1], has had a major impact on the management of patients with RA. It is now widely used in research and in many countries DAS-based thresholds determine which patients can access biologic therapy [2]. However, the DAS has limitations. Despite recommendation by the European Medicines Agency that 28-joint DAS (DAS28) remission (DAS28 < 2.6) [3] is the preferred primary outcome for trials of agents other than NSAIDs [4], there is evidence of continued radiographic progression in patients achieving this goal [5]. There is also recognition that two patients with the same DAS score can have different phenotypes [6].

The DAS was developed using rheumatologists’ treatment decisions to define periods of high and low disease activity. Of the DAS components, Ritchie articular index and 44 swollen joint count (SJC44) made the largest contribution to discriminating between these states, suggesting that the rheumatologists placed more importance on joint assessments than blood markers of inflammation (ESR) or patient subjective effects (general health visual analogue scale; GHVAS). Updated DAS definitions have since been introduced; to reduce assessment time, 28-joint counts [7] may be used. Other definitions substitute CRP for ESR [8], and omit the GHVAS [7], although these definitions are not interchangeable [9–11].

All existing DAS variants were developed using the same outcome (treatment decision) prior to the widespread use of modern imaging, which has since demonstrated disparity between clinical assessments and imaging-detected synovitis, a possible explanation for continued joint destruction in patients considered to be in clinical remission [12–17]. Baker *et al.* [18] found that of the original DAS28 components only SJC28 and acute phase reactant (CRP or ESR) were independently associated with MRI-detected synovitis, despite the DAS28 being weighted most heavily for joint tenderness. Furthermore, recent findings from the Norfolk Arthritis Register (NOAR) and Early RA Study cohorts demonstrated that HLA-DRB1 amino acids associated with RA susceptibility and radiographic joint damage were associated with SJC28 and CRP, but not 28 tender joint count (TJC28) (GHVAS was not assessed) [19]. Thus, there are biologically plausible reasons for differential associations between core DAS components and RA patient phenotypes.

In addition to SJC28 and CRP, Baker *et al.* found evaluator’s assessment of global disease activity VAS to be associated with MRI-detected synovitis. However, evaluator’s assessment of global disease activity VAS is difficult to standardize and is frequently unavailable in large-scale (e.g. genome-wide association) studies incorporating existing datasets. There is therefore a need for a short-form DAS that allows evaluation of synovitis activity in existing large international RA cohorts.

The objectives of the current study were firstly to confirm whether TJC28, SJC28, CRP, ESR and GHVAS are independently associated with imaging-detected synovitis, using US combined grey scale and power Doppler (GSPD) score as the outcome; and secondly to define a novel re-weighted DAS28 using appropriate components, and validate it against radiographic progression in an external cohort.

## Methods

All patients selected for this analysis satisfied a diagnosis of RA by 1987 ACR [20] and/or 2010 ACR/EULAR [21] criteria.

In the development phase we selected RA patient data from three sources: The Leeds Inflammatory Arthritis Continuum (IACON), a single-centre observational cohort of patients with early inflammatory arthritis recruited 2010–2014; the Pathobiology of Early Arthritis Cohort (PEAC), a multicentre observational cohort of patients with early RA recruited 2009–2015; and a clinical trial (Infliximab as Induction Therapy in Early Rheumatoid Arthritis; IDEA [22]), which was largely recruited in Leeds and satellite centres 2006–2009 – only Leeds patients are included here. Validation against radiographic progression was conducted in RA patients selected from the Norfolk Arthritis Register (NOAR), a primary care-based inception cohort of patients recruited 1989–2008, presenting with recent-onset inflammatory polyarthritis, defined as ⩾2 swollen joints for >4 weeks [23].

All patients provided written informed consent for inclusion in each of the studies, and ethical approval was granted by the following: IACON: Leeds (West) Research Ethics committee (09/H1307/98); IDEA: Northern and Yorkshire Research Ethics Committee (05/MRE03/85); PEAC: National Research Ethics Service Committee London – Dulwich (05/Q0703/198); and NOAR: Norwich Research Ethics committee (LREC 2003-075).

### Disease activity measurements

In IACON, IDEA and PEAC, clinical assessments were made independently of US assessments. Joint counts included bilateral shoulders, elbows, wrists, MCPs joints 1–5, PIPs joints 1–5, knees and MTPs joints 1–5. Patients completed a 100 mm global health assessment VAS. CRP was recorded in mg/L; for censored values (CRP < 5 mg/L), we imputed CRP = 2 prior to analysis (see ‘Statistical methods’ and ‘Results’ for more details). ESR was measured in mm/h. Observations were available in IACON at 0, 26, 52 and 104 weeks, in IDEA at 0, 52 and 78 weeks and in PEAC at 0 and 26 weeks. Visits were eligible if the US scan occurred within 1 week of the clinical assessment.

In NOAR CRP was measured in stored serum samples collected at 0, 5, 10 and 15 years; consequently, DAS28CRP was only calculated at these time points. Tender and SJCs were carried out at 0, 3, 5, 10, 15 and 20 years. A three-component (3C) DAS28 score was calculated for comparison with the re-weighted DAS28, as patient GHVAS was not available.

### Ultrasound

Full details of US scanning procedures are provided in online Supplementary material, section ‘Methods’, available at *Rheumatology* online. In IACON and IDEA grey scale (GS) and power Doppler (PD) synovitis were each scored semi-quantitatively 0–3 [12]; GS scores included both synovial hyperplasia and joint effusion. Sagittal plane views were scored. In IACON, scoring was performed by several trained sonographers and rheumatologists. In IDEA the majority of scoring was performed by one expert rheumatologist. In IDEA and IACON the following joints were scanned: wrists, MCPs 2–3, PIPs 2–3, knees, MTPs 1–5. In PEAC GS and PD were scored from 0–4 against a standardized image atlas [24], using transverse plane views; bilateral MCPs 1–5 were scanned.

### Radiography

In NOAR, radiographs of the hands and feet were performed during the first 10 years of follow-up and were scored using the Larsen method [25] as previously described [26–28]. Joint erosion was defined as a cortical break of ⩾2 mm and was assigned a score of ⩾2. Radiographs were read independently, blind to sequence, by 2 medically qualified observers (intra- and inter-observer agreement for erosion presence 90% and 91%, respectively), who underwent specific training. Disagreements on erosion status were arbitrated by a third investigator.

### Statistical methods

In IACON and IDEA, OMERACT-EULAR composite PDUS scores that combined GS and PD at the joint level [29] were summated across 22 joints to give GSPD. 

In PEAC, GS and PD scores were simply summated across 10 joints, to give GSPD.

In the largest dataset (IACON), within observed data, robust regression on order statistics was used to impute left-censored values of CRP < 5 mg/L; summary statistics calculated for the imputed values indicated which single value should be imputed in the main analysis. Single imputation was chosen over model-based imputation to provide clarity for clinicians and researchers wishing to use the new score. The DAS28-CRP was originally derived using high sensitivity (hs)CRP; however, it is often calculated at centres where the reporting limit is 5, leading to variation in the values imputed for CRP < 5 mg/L.

Multiple imputation by chained equations was used to address missing covariate data; GSPD was not included in covariate imputation models and only patients with observed GSPD were retained. Imputation models included DAS28 components, transformed where appropriate to maintain consistency with analysis models [√SCJ28, √TJC28, ln(ESR), ln(CRP+1), GHVAS], Health Assessment Questionnaire Disease Index (HAQ-DI) score, age and sex. Predictive mean matching was used to impute all variables; results from 20 imputed datasets were combined according to Rubin’s rules.

Conventional DAS28 scores were calculated as follows (CRP mg/L, GHVAS mm):

4C-DAS28CRP = (0.56 × √TJC28) + (0.28 × √SJC28) + (0.36 × ln(CRP+1)) + (0.014 × GHVAS) + 0.96

4C-DAS28ESR = (0.56 × √TJC28) + (0.28 × √SJC28) + (0.70 × ln(ESR)) + (0.014 × GHVAS)

3C-DAS28CRP = [(0.56 × √TJC28) + (0.28 × √SJC28) + (0.36 × ln(CRP+1))] × 1.10 + 1.15

In addition we calculated partial simplified disease activity index (SDAI) clinical disease activity index (CDAI) scores, which excluded physician VAS, as follows (CRP mg/dL, GHVAS cm):

Partial SDAI = TJC28 + SJC28 + CRP + GHVAS

Partial CDAI = TJC28 + SJC28 + GHVAS

We modelled GSPD as a function of individual DAS28 components in linear mixed models, using 20-fold cross-validation to evaluate predictive performance on data not seen before; multiple imputation was not nested within training folds. Predictor performance was evaluated as the squared Pearson correlation (*R*^2^) between predicted and observed US scores, concatenating all test folds into a single dataset. The strength of evidence favouring one model over another was evaluated as the difference in test log-likelihoods. For each individual in the test dataset, the log of the multivariate Gaussian likelihood of the model given the residuals was calculated, and this test log-likelihood was summed over all individuals in all test folds. The difference in test log-likelihood can be interpreted directly as a measure of the strength of evidence favouring one model over another. The asymptotic equivalence of model choice by leave-one-out cross-validation and Akaike’s information criterion (AIC) [30] implies that a difference in test log-likelihood of 2.6 natural log units is equivalent (for comparison of linear nested models differing by two extra parameters) to *P* = 0.01.

To account for differences in scaling of the GSPD outcome, ratios between coefficients obtained from each cohort were weighted by the number of cases with non-missing acute phase measurements to produce the final definition for the re-weighted DAS28. For example, the ratio of the coefficient for SJC28 to the coefficient for CRP from each cohort was multiplied by the number of observations, then the values from the three cohorts were added together and divided by the total number of observations to give the final ratio to be used in the score. We did not attempt to scale the final score to maintain compatibility with the conventional DAS28 definition(s) because they were derived using entirely different methods and existing cut-offs for remission or severity of disease activity would not be valid for use with the new score.

As previously described [19, 28, 31], the Larsen score was modelled as a longitudinal continuous non-normally distributed outcome variable using a generalized linear latent and mixed model [32] that included the covariates age and disease duration in addition to disease activity variables. Multivariable models including individual DAS28 components [√TJC28, √SJC28, ln(CRP+1)] were constructed first, followed by models that included each DAS28 score (re-weighted 2C or conventional 3C) individually. Effect sizes are given as a β-coefficient with 95% CIs. AIC and Bayesian information criteria were used to determine model fit; lower values indicate better fit. The presence of erosions of the hands and feet was treated as a longitudinal binary variable and modelled using a generalized estimating equation model with logit-link function and an exchangeable within-subject correlation structure. The quasi-AIC, the equivalent of AIC in a generalized estimating equation, was used to determine model fit [33].

Analyses that included US were conducted in R version 3.3.1 (R Foundation for Statistical Computing, Vienna, Austria); analyses of radiographic outcomes were carried out in Stata v14 (StataCorp, College Station, TX, USA).

## Results

Baseline characteristics of patients are provided in Table 1. We included 889 observations in 434 IACON patients, 163 in 59 IDEA patients, and 183 in 117 PEAC patients who had at least one US measurement. For validation against radiographic progression there were 3037 observations in 717 NOAR patients.

**Table 1:** Baseline characteristics of patients included in IACON, IDEA, PEAC and NOAR

	IACON	IDEA	PEAC	NOAR
	(*n* = 434)	(*n* = 59)	(*n* = 117)	(*n* = 717)
Age, mean (s.d.), years	56 (15)	51 (12)	51 (16)	58 (14)
Female, % (*n*)	70 (304/434)	72 (50/69)	68 (80/117)	69 (492/717)
Symptom duration, months	7 (4, 15)	7 (5, 10)	5 (3, 8)	8 (4, 17)
RF positive, % (*n*)	63 (245/392)	57 (39/68)	74 (86/116)	32 (209/644)
ACPA positive, % (*n*)	69 (300/432)	74 (48/65)	81 (95/117)	38 (276/717)
TJC28	4 (1, 10)	15 (8, 22)	10 (5, 15)	5 (1, 11)
SJC28	2 (0, 5)	8 (3, 11)	5 (3, 8)	5 (2, 9)
CRP, mg/L	6 (<5, 18)	21 (10, 52)	7 (<5, 18)	11 (3, 24)
ESR, mm/h	23 (10, 38)	37 (19, 65)	30 (15, 49)	—
GHVAS, mm	35 (15, 57)	53 (39, 72)	72 (51, 84)	—
GS^a^	15 (9, 22)	29 (19, 35)	15 (8, 22)	—
PD^a^	2 (0, 6)	13 (8, 20)	2 (4, 9)	—
Total Larsen score^b^	—	—	—	5 (0, 19)
Erosions present, % (*n*)	—	—	—	45 (325/717)

Values presented are median (1st quartile, 3rd quartile) unless otherwise stated. The table provides baseline demographic, clinical and imaging characteristics of patients included in the study. ^a^Different sets of joints were scanned in IACON/IDEA compared with PEAC, and therefore the US scores are not directly comparable across all cohorts. ^b^Total of individual 0–5 scores for joints in the hands and feet. GHVAS: general health visual analogue scale; GS: grey scale; PD: power Doppler; SJC28: 28 swollen joint count; TJC28: 28 tender joint count; IACON: The Leeds Inflammatory Arthritis Continuum; IDEA: Infliximab as Induction Therapy in Early Rheumatoid Arthritis; PEAC: Pathobiology of Early Arthritis Cohort; NOAR: Norfolk Arthritis Register.

### Imputation of CRP < 5

The median of values imputed in IACON using robust regression on order statistics for values of CRP < 5 was 1.88. We therefore imputed 2 for observations of CRP < 5. See Table 2 for numbers of cases in which this was necessary.

**Table 2:** Availability of DAS28 component data within each cohort included in the development phase

	IACON	IDEA	PEAC	NOAR
Longitudinal observations, *n*	889	163	183	3037
Missing TJC28, *n*	3	0	22	0
Missing SJC28, *n*	3	0	2	0
Missing GHVAS	59	0	2	NA
Missing ESR, *n*	159	10	3	NA
Missing CRP, *n*	53	9	5	135
CRP values censored (<5 mg/L), *n*	431	72	51	0^a^

The table shows the total number of observations available in each of the included cohorts and the extent of missing and censored data. ^a^High-sensitivity CRP was collected in NOAR. GHVAS: general health visual analogue scale; SJC28: 28 swollen joint count; TJC28: 28 tender joint count; IACON: The Leeds Inflammatory Arthritis Continuum; IDEA: Infliximab as Induction Therapy in Early Rheumatoid Arthritis; PEAC: Pathobiology of Early Arthritis Cohort; NOAR: Norfolk Arthritis Register; NA: Not available.

### Associations between DAS28 components and GSPD

Associations between DAS28 components and GSPD were only investigated in IACON, IDEA and PEAC; NOAR was excluded to provide external validation of the new scale against radiographic progression.

In four-component (4C) models that included CRP, in all three cohorts only SJC28 and CRP were independently associated with GSPD. Results for models with two or four individual components, using either CRP or ESR, are presented in Table 3.

**Table 3:** Coefficients for regression of global GSPD combined score onto individual DAS28 components in three datasets

	**Coefficient** ^a^ **(95% CI)**					
	**Four-component model**			Two-component model		
	IACON	IDEA	PEAC	IACON	IDEA	PEAC
With CRP						
Intercept	85.44	63.28	−0.48	84.32	66.74	−0.78
	(71.92, 98.96)	(32.40, 94.15)	(−4.23, 3.27)	(71.97, 96.66)	(37.66, 95.81)	(−4.18, 2.63)
ln(CRP+1)	15.79	39.64	4.28	15.45	40.31	4.06
	(9.95, 21.62)	(23.39, 55.89)	(2.65, 5.92)	(9.79, 21.14)	(24.78, 55.84)	(2.46, 5.66)
√SJC28	30.48	25.70	4.54	30.61	31.40	4.69
	(24.49, 36.47)	(6.04, 45.36)	(2.58, 6.49)	(25.91, 35.31)	(18.33, 44.47)	(3.34, 6.03)
√TJC28	0.62	6.72	0.70			
	(−4.43, 5.67)	(−9.57, 23.01)	(−1.00, 2.40)			
GHVAS	-0.07	-0.06	-0.04			
	(−0.32, 0.17)	(−0.71, 0.60)	(−0.10, 0.02)			
With ESR						
Intercept	79.47	87.73	1.69	79.33	96.81	1.49
	(69.90, 99.04)	(44.15, 131.30)	(−2.95, 6.32)	(60.12, 98.54)	(54.93, 138.70)	(−2.97, 5.94)
ln(ESR)	11.91	10.68	1.54	11.74	12.68	1.43
	(4.87, 18.94)	(−6.53, 27.88)	(−0.22, 3.31)	(4.91, 18.57)	(−4.32, 29.67)	(−0.28, 3.13)
√SJC28	32.08	35.41	5.66	32.30	47.70	5.59
	(26.06, 38.09)	(14.21, 56.60)	(3.59, 7.74)	(27.57, 37.02)	(34.39, 61.00)	(4.08, 7.10)
√TJC28	0.49	10.11	0.15			
	(−4.61, 5.58)	(−7.21, 27.43)	(−1.64, 1.95)			
GHVAS	−0.03	0.25	−0.02			
	(−0.28, 0.22)	(−0.43, 0.94)	(−0.09, 0.05)			

Coefficients in this table are from linear mixed regression models containing individual DAS28 components; on the left are results for models containing all four components; on the right are results for models containing only SJC and acute phase (CRP or ESR). ^a^Different sets of joints were scanned in IACON/IDEA compared with PEAC, and different methods were used to calculate global GSPD combined score, and therefore the coefficients are not directly comparable across all cohorts. GHVAS: general health visual analogue scale; GSPD: grey scale and power Doppler; SJC28: 28 swollen joint count; TJC28: 28 tender joint count; IACON: The Leeds Inflammatory Arthritis Continuum; IDEA: Infliximab as Induction Therapy in Early Rheumatoid Arthritis; PEAC: Pathobiology of Early Arthritis Cohort; NOAR: Norfolk Arthritis Register.

Repeating this analysis with complete case data, within each cohort, did not alter the conclusions (data not shown).

### Evaluation of model fit to GSPD

In all three cohorts, both *R*^2^ and test log-likelihood values favoured the two-component (2C) model that included only SJC28 and the acute phase reactant over the models containing all four components of conventional DAS28 (Table 4). We explored adding both CRP and ESR into the same model as SJC28; the 2C CRP model was still consistently a better fit to GSPD in terms of *R*^2^, although test log-likelihood for this model was slightly higher in PEAC.

**Table 4:** Comparison of predictions from models with varying disease activity components in test data by cross-validation

Model	*R* ^2^ between observed and predicted values in test data			Difference in test log-likelihood (natural log units) from best model		
	IACON	IDEA	PEAC	IACON	IDEA	PEAC
Individual component models						
ln(CRP) + √SJC28	0.236	0.392	0.380	0	0	0
ln(CRP) + ln(ESR) + √SJC28	0.232	0.384	0.378	−4.58	−4.85	0.41
ln(CRP) + √SJC28 + √TJC28 + GHVAS	0.232	0.380	0.373	−1.85	−3.04	−1.59
ln(ESR) + √SJC28	0.215	0.309	0.306	−19.77	−7.55	−9.19
ln(ESR) + √SJC28 + √TJC28 + GHVAS	0.211	0.298	0.297	−22.62	−12.31	−10.48
DAS models						
Re-weighted 2C-DAS28CRP	0.236	0.386	0.383	0	0	0
Conventional 3C-DAS28CRP	0.166	0.356	0.295	−55.52	−5.73	−15.39
Conventional 4C-DAS28CRP	0.144	0.337	0.256	−78.21	−10.69	−18.85
Conventional 4C-DAS28ESR	0.123	0.305	0.222	−102.03	−7.42	−20.31
Partial SDAI	0.166	0.337	0.251	−62.64	−8.82	−17.62
Partial CDAI	0.139	0.295	0.209	−78.12	−13.95	−20.89

This table provides the squared Pearson correlation (*R*^2^) for a variety of different linear mixed models each predicting global GSPD combined score, including two, three or four of the original individual DAS28 components, or a single calculated DAS; the difference in test log-likelihood (a measure of goodness-of-fit to the outcome) is also provided for each model relative to the best-performing model (for which difference = 0). 2C: two component; 3C: three component; 4C: four component; CDAI: clinical disease activity index; DAS28: DAS using 28 joint counts; GHVAS: general health visual analogue scale; GSPD: grey scale and power Doppler; SDAI: simplified disease activity index; SJC28: 28 swollen joint count; TJC28: 28 tender joint count; IACON: The Leeds Inflammatory Arthritis Continuum; IDEA: Infliximab as Induction Therapy in Early Rheumatoid Arthritis; PEAC: Pathobiology of Early Arthritis Cohort; NOAR: Norfolk Arthritis Register.

ESR was not independently associated with GSPD in IDEA or PEAC. The 2C ESR model was a better fit than the 4C ESR model in all three cohorts. However, the 2C CRP model was consistently a better fit than the 2C ESR model. Therefore, we consider the 2C CRP-based model the most appropriate measure of global synovitis.

### Derivation of re-weighted DAS28

Ratios for SJC28: CRP from the 2C models were 1.98 (IACON), 0.78 (IDEA) and 1.16 (PEAC). Weighting for the number of non-missing CRP observations (Table 2), the combined ratio was 1.68. This yielded the following 2C-DAS28CRP equation (imputing 2 if CRP is censored at <5mg/L):

2C−DAS28CRP=√SJC28+(0.6×ln(CRP+1))

We then calculated DAS28 according to conventional definitions (3C-DAS28CRP, 4C-DAS28CRP and 4C-DAS28ESR), and according to the new 2C-DAS28CRP defined above, in addition to partial SDAI and partial CDAI. *R*^2^ values for the association with GSPD were considerably higher for 2C-DAS28CRP than for the conventional scores (Table 4), and large differences in test log-likelihood confirmed that the new score outperformed the existing definitions.

While we consider 2C-DAS28CRP the most appropriate measure of global synovitis, to permit analysis of historical datasets with only ESR available we constructed 2C-DAS28ESR, using the same method to combine SJC28: ESR coefficient ratios across the three cohorts:

2C−DAS28ESR =√SJC28 + (0.32 × ln(ESR))

### Associations between DAS28 components and radiographic outcome

Results of analyses testing the association of DAS28 components with radiographic outcomes are presented in Table 5. TJC28 was not associated with Larsen score, while SJC28 and CRP were both positively associated. Higher TJC28 was associated with lower odds of erosion, while SJC28 and CRP were both positively associated with erosion.

**Table 5:** Associations between individual DAS28 components and radiographic damage

	Univariable models		Multivariable model	
Variable	β-Coefficient (95% CI)	*P*-value	β-Coefficient (95% CI)	*P*-value
Larsen score (GLLAMM)				
√TJC	−0.21 (−0.47, 0.05)	0.111	−0.48 (−1.04, 0.08)	0.092
√SJC	0.67 (0.37, 0.98)	1.31 × 10^−5^	0.84 (0.13, 1.55)	0.020
ln(CRP+1)	1.65 (1.09, 2.22)	9.85 × 10^−9^	1.65 (1.07, 2.23)	2.29 × 10^−8^
Erosions (GEE)				
√TJC	−0.13 (−0.18, -0.08)	2.30 × 10^−7^	−0.28 (−0.40, −0.16)	2.52 × 10^−6^
√SJC	0.06 (−0.00, 0.11)	0.064	0.30 (0.15, 0.44)	5.21 × 10^−5^
ln(CRP+1)	0.30 (0.21, 0.40)	6.55 × 10^−10^	0.31 (0.20, 0.42)	5.31 × 10^−8^

This table provides coefficients, 95% CI and *P*-values for univariable and multivariable longitudinal models of radiographic progression that include individual components of conventional three-component DAS28CRP. DAS28: DAS using 28 joint counts; GEE: generalized estimating equations; GLLAMM: generalized linear latent and mixed models; SJC: swollen joint count; TJC: tender joint count.

### Comparison between conventional and re-weighted DAS28 scores

Conventional 3C-DAS28CRP was significantly associated with Larsen score; however, the association was stronger with re-weighted 2C-DAS28CRP (Table 6). Furthermore, model fit was improved with the re-weighted 2C-DAS28CRP score.

**Table 6:** Comparison of associations between radiographic damage and conventional *vs* re-weighted DAS28

Model	β-Coefficient (95% CI)	*P*-value	AIC	BIC	QIC
Larsen score					
Conventional 3C-DAS28CRP	0.78 (0.27, 1.30)	0.003	8140.085	8213.550	
Re-weighted 2C-DAS28CRP	1.15 (0.69, 1.61)	8.68 × 10^−7^	8124.842	8198.308	
Erosions					
Conventional 3C-DAS28CRP	0.07 (−0.03, 0.17)	0.174			1611.693
Re-weighted 2C-DAS28CRP	0.24 (0.15, 0.33)	2.02 × 10^−7^			1545.371

This table provides coefficients, 95% CI, *P*-values and model fit statistics for univariable longitudinal models of radiographic progression that include either the re-weighted 2C- or conventional 3C-DAS28CRP score. 2C: two component; 3C: three component; AIC: Akaike information criteria; BIC: Bayesian information criteria; DAS28: DAS using 28 joint counts; QIC: quasi-AIC.

Conventional 3C-DAS28CRP was not associated with the presence of erosions. However, re-weighted 2C-DAS28CRP was significantly associated with the presence of erosions. Model fit was again better with the re-weighted 2C-DAS28CRP score.

## Discussion

This is the first study to produce a 2C DAS weighted against US-detected inflammation and demonstrate that it outperforms the conventional definitions in the strength of association both with synovitis and radiographic progression. The resulting 2C-DAS28CRP is a potential tool for assessing one pathophysiological manifestation of RA, i.e. synovitis, consistent with the OMERACT core set definition [34].

RA DASs were intended to capture the severity of the patient’s inflammatory symptoms. The 2C-DAS28CRP was developed to better reflect the pathophysiological manifestation of synovial inflammation and not a more global construct of disease activity, as reflected in the breadth of the OMERACT core set. Consequently, we would not recommend replacing the conventional DAS28 with the 2C score in clinical trials or clinical practice without due consideration of how to measure the other core set areas. The 2C-DAS28CRP score will therefore be useful in studies that focus on the pathophysiological manifestations of RA: these include (pharmaco)genetic/genomic studies. It could be argued that to identify biological factors associated with disease activity or response to therapy, US inflammation should be used directly as the outcome. This would have the advantage of allowing us to separate synovitis from tenosynovitis, although it is likely that these both fall into a broader construct of ‘RA inflammation’ which would remain an appropriate target for such research. However, it is not currently possible to acquire and score images in routine clinical settings, from which most of the large (pharmaco)genetic/genomic datasets have been compiled to date and are likely to be compiled in the near future. The 2C-DAS28CRP will therefore aid the (re)analysis of (existing) large-scale datasets and may yield larger effect sizes for any putative associations in comparison with existing DAS28CRP.

Our findings show that if TJC28 and patient GHVAS are removed from the DAS28, the association with the pathophysiological mechanism targeted by DMARDs, namely synovial inflammation, is improved. The association was stronger for CRP-based models than for ESR-based models, consistent with CRP being a more sensitive and responsive measure of inflammation than ESR, which is an indirect measure of multiple plasma proteins and influenced by age, gender, haemoglobin and serum immunoglobulin levels, among other confounders [35].

In previous work Baker *et al.* [18] found that SJC28, acute phase reactants and evaluator’s global assessment were associated with MRI-detected synovitis, which broadly agrees with our findings. However, we were unable to investigate the association with physician’s global assessment as this was not available in our cohorts; indeed, this partly motivated the development of the new score. For the same reason we were unable to calculate full SDAI and CDAI scores; however, the equivalent partial scores performed poorly in comparison with 2C-DAS28CRP, because they gave equal weight to tender and swollen joints and included patient VAS, when neither tenderness nor patient VAS were independently associated with GSPD.

In a previous attempt to update the DAS28 using US-detected synovitis [36], TJC28 and SJC28 were replaced with PD score from 22 joints (including MTPs and excluding elbows, shoulders and PIPs), and a count of GS synovitis presence in the standard 28 joints. However, coefficients for each term were not amended, and replacing the clinical counts with US restricts the clinical utility of the score because it requires US assessment in all cases. Nevertheless, the authors reported stronger associations between their US-derived DAS28 scores and radiographic and MRI measures of structural progression compared with the original DAS28.

There are a number of limitations to this study. A limited amount of missing covariate data was addressed using multiple imputation, which has been shown to reduce imprecision and bias in the estimation of coefficients in comparison to *ad hoc* approaches such as complete case analysis [37]. We did not collect hsCRP and therefore opted to impute a single value for observations of CRP < 5 to standardize practice when using the new scale. It is possible that for patients with no swollen joints, hsCRP would provide valuable additional information, and a DAS using alternative weights may be more appropriate in such circumstances. However, hsCRP is not routinely available in the clinic, limiting application. US assessments of different joint sets were made for IACON/IDEA and PEAC, and the methods of calculating GSPD differed between the cohorts. Coupled with potential case mix differences, this explains why intercepts and coefficients differed between the cohorts, which we addressed by using ratios of the coefficients rather than the coefficients themselves. The inclusion of cohorts representing a spectrum of disease activity and treatment is desirable in a validation study. Despite the differences in case mix evident in Table 1, and the differences in US methodology, patterns of association between DAS28 components and the US outcome were the same across the development cohorts. The validation cohort was recruited prior to the introduction of rapid DMARD escalation for management of RA; therefore, the level of radiographic progression may have been higher than we would expect in modern cohorts. However, greater range in the outcome is desirable when studying associations and, importantly, we identified the same patterns of association, this time replacing US inflammation with its posited consequence, radiographic progression, as the outcome. We chose not to rely on observed coefficients so our results would be widely generalizable across varied cohorts. Furthermore, any differences between cohorts could not have affected the finding that 2C-DAS28CRP was more strongly associated with the outcome than original DAS28 within each cohort. Study design is an important consideration when US methods are not standardized; it would not be appropriate to directly compare levels of inflammation between cohorts such as the ones included in this study. However, our results show that with the use of carefully selected methods (such as using ratios rather than measured coefficients) it is possible to derive clinically meaningful results by combining data from a range of US scoring systems. In future, advances in machine learning for feature extraction and scoring may help improve standardization. GS scoring may reflect fibrosis in addition to inflammation; we have only included patients with early RA in this development study, which should limit the impact of this issue, but may also limit the applicability of the scale for use in patients with more established disease. Future areas of validation work for the new scale will include reassessment of treatment effects in historical trials of both early and established RA, in addition to prospective data collection.

In summary, a re-weighted DAS28 equation including only SJC28 and CRP was more closely associated with US-detected synovitis than definitions that also included TJC28 and GHVAS. CRP-based DASs were more closely associated with synovitis than ESR-based counterparts. The 2C-DAS28CRP showed stronger association with burden of radiographic damage than the conventional 3C-DAS28 including TJC28. The improved association with both synovitis and erosion demonstrates that the novel 2C-DAS28 is a more appropriate measure of pathophysiology in early RA compared with conventional DAS28.

## Supplementary Material

kez049_Supplementary_Data
