# Supplementary material for: Validity of a two-component imaging-derived disease activity score for improved assessment of synovitis in early rheumatoid arthritis
Source: Rheumatology (Oxford). 2019 Mar 1;58(8):1400–9. doi: 10.1093/rheumatology/kez049 (PMC6649844; doi:10.1093/rheumatology/kez049)
Supplement: kez049_Supplementary_Data [file kez049_supplementary_data.docx]

**SUPPLEMENTARY MATERIAL.**

**Methods**

**Ultrasound assessments**

In IDEA ultrasound (US) scans were performed using a Philips (ATL HDI 5000) machine with 5–12 and 8–15 MHz transducers. Power Doppler frequency (PDF) was 6 MHz; PD was assessed using a pulse repetition frequency (PRF) set between 700-1000 MHz, medium wall filter and gain adjusted until the background noise was suppressed. In IACON, a General Electric E9 machine was used with 15-6 and 18–8 MHz linear array transducers. The scanning parameters were: B mode frequency (12–18 MHz), B mode gain 44–54 db, PDF 7.5–10 MHz, PRF 800 Hz (0.8 kHz) and wall filter low–medium.

In PEAC US scans were performed using a General Electric Logiq 9 machine. For grey scale (GS) frequency was set at 14 MHz, gain 50. For power Doppler frequency was 7.5 MHz, gain 41, wall filter 127 Hz.

In IACON the majority of US assessments were performed by one of two full-time sonographers who performed n=342 and n=250 assessments respectively. Of the remainder, n=68 and n=48 assessments were performed by two rheumatologists with extensive musculoskeletal ultrasonography experience. The identity of the person performing the assessment was not recorded for n=129 assessments. The remaining 52 assessments were performed by one of eight rheumatologists trained in musculoskeletal ultrasound.

In IDEA US assessments were performed by one of four rheumatologists trained in musculoskeletal ultrasound; the majority of assessments (80%) were carried by one individual with extensive musculoskeletal ultrasonography experience.

In PEAC US scoring was performed by two readers for whom single-measure inter-reader intraclass correlation coefficients (ICCs) for synovial thickness (GS) and PD were 0.768 (0.367, 0.931) and 0.965 (0.936, 0.980) respectively (using SPSS v21.0 for Mac).

**Consortia members**

**MATURA Consortium**

**Work Stream 1**: Prof Costantino Pitzalis, Queen Mary University of London, Prof Peter Taylor, University of Oxford, Prof Ernest Choy, Cardiff University, Prof Iain McInnes, University of Glasgow, Dr Mike Barnes, Queen Mary University of London, Prof John Isaacs, Newcastle University, Prof Christopher Buckley, University of Birmingham, Prof Michael Ehrenstein, University College London, Prof Peter Sasieni, Queen Mary University of London, Dr Andrew Filer, University of Birmingham

**Work Stream 2:** Prof Anne Barton, University of Manchester, Prof Ann Morgan, University of Leeds, Prof Gerry Wilson, University College Dublin, Prof Paul McKeigue, University of Edinburgh, Prof Heather Cordell, Newcastle University, Prof Jenny Barrett, University of Leeds, Prof Andrew Cope, Kings College London, Prof Adam Young, University of Hertfordshire, Prof Karim Raza University of Birmingham, Prof Katherine Payne University of Manchester, Prof Jane Worthington University of Manchester, Prof Deborah Symmons University of Manchester, Prof Kimme Hyrich University of Manchester Industry Martin Hodge – Pfizer Anthony Rowe- Janssen, Jianmei Wang -Roche/Genentech, Michelle Mao BGI, Patricia McLoughlin –Qiagen, Carolyn Cuff – AbbVie, David Close- MedImmune

**IACON Consortium**

University of Leeds and Leeds Teaching Hospitals NHS Trust

**IACON Management Team:** Paul Emery, Jane Freeston, Elizabeth Hensor

**IACON Consultant and Senior Scientific Staff:** Ai Lyn Tan, Jackie Nam, Philip Conaghan, Ann Morgan, Frederique Ponchel, Maya Buch, Jacqueline Andrews, Richard Wakefield, Denis McGonagle, Helena Marzo-Ortega, Aamir Aslam, Ed Vital

**IACON Specialist Registrars and Clinical Fellows:** Edith Villeneuve, Sudipto Das, Sarah Horton, Sarah Mackie, Rebecca Thomas, Lesley Ann Bissell, Chadi Rakieh, Zoe Ash, Sarah Twigg, Laura Coates, Fahad Fazal, Laura Hunt, Esme Ferguson, Sara Else, Gui Tran, Ahmed Zayat, Giuseppina Abignano, Md Yuzaiful Md Yusof, Radhika Ragunath, Hannah Mathieson, Chitra Salem-Ramakumaran, Hanna Gul, Mahwish Mahmood

**IACON Nursing Staff:** Pauline Fitzgerald, Matthew Robinson, Jason Ward, Beverly Wells, David Pickles, Oliver Wordsworth, Christine Thomas, Alison McManus, Lynda Bailey, Linda Gray, Kate Russell, Jayne Davies

**IACON Laboratory and Support Staff:** Diane Corscadden, Karen Henshaw, Stephen Martin, James Robinson, Agata Burksa, Sarah Fahy, Jill Halsted-Rastrick, Ged Connolly-Thompson, Jonathan Thompson, Ian Weatherill, Andrea Paterson

**IACON Radiology team:** Laura Horton, Alwyn Jackson, Richard Hodgson

**PEAC Consortium**

Costantino Pitzalis

Iain McInnes

Peter Taylor

Chris Buckley

Ernest Choy
